# Interactions between plants and soil shaping the root microbiome under abiotic stress

**DOI:** 10.1042/BCJ20180615

**Published:** 2019-10-11

**Authors:** Kyle Hartman, Susannah G. Tringe

**Affiliations:** 1 U.S. Department of Energy Joint Genome Institute, Walnut Creek, CA 94598, U.S.A.; 2 Environmental Genomics and Systems Biology Division, Lawrence Berkeley National Laboratory, Berkeley, CA 94720, U.S.A.

**Keywords:** abiotic stress, host–microbe interactions, root microbiome

## Abstract

Plants growing in soil develop close associations with soil microorganisms, which inhabit the areas around, on, and inside their roots. These microbial communities and their associated genes — collectively termed the root microbiome — are diverse and have been shown to play an important role in conferring abiotic stress tolerance to their plant hosts. In light of growing concerns over the threat of water and nutrient stress facing terrestrial ecosystems, especially those used for agricultural production, increased emphasis has been placed on understanding how abiotic stress conditions influence the composition and functioning of the root microbiome and the ultimate consequences for plant health. However, the composition of the root microbiome under abiotic stress conditions will not only reflect shifts in the greater bulk soil microbial community from which plants recruit their root microbiome but also plant responses to abiotic stress, which include changes in root exudate profiles and morphology. Exploring the relative contributions of these direct and plant-mediated effects on the root microbiome has been the focus of many studies in recent years. Here, we review the impacts of abiotic stress affecting terrestrial ecosystems, specifically flooding, drought, and changes in nitrogen and phosphorus availability, on bulk soil microbial communities and plants that interact to ultimately shape the root microbiome. We conclude with a perspective outlining possible directions for future research needed to advance our understanding of the complex molecular and biochemical interactions between soil, plants, and microbes that ultimately determine the composition of the root microbiome under abiotic stress.

## Introduction

**Figure 1.**
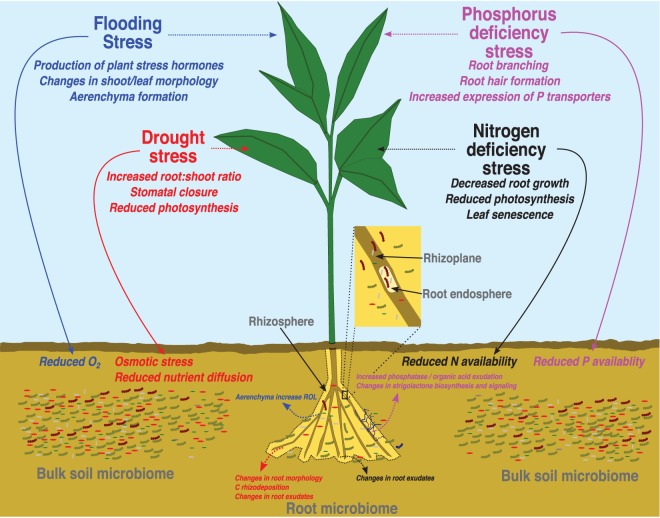
Abiotic stress factors shaping the root microbiome. Overview of direct effects of selected abiotic stress factors on soil (solid lines with arrows) that shape the pool of microbes colonizing the different compartments of root microbiome (labeled in gray and indicated with arrows). Dashed lines represent indirect effects of the abiotic stress factors on the host plant that subsequently influence root microbiome assembly under the different abiotic stress conditions.

As sessile organisms, plants are subjected to a constant barrage of biotic and abiotic stressors. Because their survival depends on their ability to respond to stress, plants have evolved a variety of mechanisms to mitigate its negative impacts. One such mechanism involves capitalizing on the close association between a plant's roots and specific groups of soil microbes with plant-beneficial properties. These plant–microbe associations occur at the root–soil interface known as the rhizosphere — a term first defined by Hiltner in 1904 as the area of soil under the biochemical influence of plant roots [1]. Although there is no definitive spatial boundary of the rhizosphere, it has been proposed to extend 1–5 mm from the root surface into the surrounding soil (Figure 1) [2]. The root surface and inner root tissues are also colonized by microbes — microhabitats termed the rhizoplane [3,4] and root endosphere [5,6], respectively (Figure 1). Together, the microbes that inhabit these environments and their associated genes collectively function as the root microbiome, and bacteria, archaea, fungi, and oomycetes are all known colonizers of plant roots [7,8]. The larger, more diverse bulk soil microbial community is the starting pool from which plants actively recruit microbes to their roots. Thus, the most influential factors determining the composition of root bacterial and fungal communities are the composition of the larger starting pool of soil microbes and the individual root compartment — i.e. the rhizosphere, rhizoplane, or root endosphere [9–15]. Edaphic factors like pH have been identified as major drivers of bulk soil bacterial [16–18] and fungal [19] community composition, but differences in availability and stoichiometry of nutrients including phosphorus (P), nitrogen (N), and carbon (C) also play an important role [17]. As these edaphic factors influence the community composition of bulk soil microbial communities, so too do plants exert influence on their root microbiome [20]. Assembly and composition of a plant's rhizosphere community is mediated by exudation of complex C compounds, mucilage, sloughed root border cells, and microbe signaling hormones, which creates nutrient-rich conditions for microbial growth and acts as a first selection step to attract a subset of soil microbes to the rhizosphere [20–23]. In a second step, host-plant genetic factors mediate the production of different microbe signaling compounds and activation of plant immune responses that permit a subset of microbes from the rhizosphere to bind to the rhizoplane and, subsequently, a subset of the rhizoplane community to enter and proliferate in the root endosphere [20,23].

The bacterial and fungal members of the root microbiome can establish commensal, pathogenic, and beneficial associations with their host [24,25], as well as with each other [26]. A large body of evidence highlights the beneficial services provided by the root microbiome, particularly its importance in maintaining plant productivity by contributing to plant biotic and abiotic stress resistance and resilience via many mechanisms [24,27,28]. These include plant growth-promoting (PGP) services like direct and indirect antagonism of plant pathogens via antibiotic production and resource competition, fixation and solubilization of growth-limiting nutrients, activation of plant immune system responses, and direct growth promotion via the production of plant growth hormones [23,29,30]. One well-known PGP group of microbes is bacteria, primarily from the family *Rhizobiaceae*, that form endosymbiotic associations with leguminous plants by inhabiting root nodules. Here, they are capable of biological nitrogen fixation (BNF) — the transformation of atmospheric N_2_ gas into bioavailable N [31], a limiting nutrient in most ecosystems [32]. Other bacteria from diverse taxonomic lineages capable of BNF have also been found in the rhizospheres of non-legumes, including many agriculturally relevant plant species [33]. In addition to N fixation, bacteria have been shown to be able to solubilize and mineralize inorganic and organic pools of soil P and transform it into plant-available phosphate, thereby increasing P uptake and growth in a variety of plant species [34]. Many fungi also demonstrate PGP properties [35] and have been shown to solubilize P [36] and increase N uptake in their associated plant hosts [37]. Perhaps the most studied group of PGP fungi is the arbuscular mycorrhizal fungi (AMF), which belong to the Glomeromycota and form symbiotic associations with ∼80% of terrestrial plant species by colonizing the root endosphere [38]. In exchange for plant-derived C, AMF hyphae extend into the soil, accessing available phosphate in soil microsites, and subsequently transfer it to their host. It has been estimated that AMF can contribute up to 70–90% of plant P [39,40] which can substantially improve plant growth under low P conditions [38]. Plant colonization by AMF has also been linked to increased N uptake [41] and enhanced drought tolerance [42]. Taken together, these examples suggest that there is considerable potential to utilize specific members of the root microbiome to increase plant tolerance to abiotic stress conditions.

In the future, terrestrial ecosystems, particularly those vital to global food and bioenergy production, are expected to face increasing abiotic stress in the form of periods of flooding and drought due to changing precipitation patterns under global climate change [43,44], changes in availability of nutrients critical to plant growth due to intensive land use and increasing fertilizer application, and projected declines in nutrient use efficiency and fertilizer stocks [45–47]. Given the aforementioned beneficial services provided to plants by their microbial symbionts, understanding how these root communities respond to such environmental changes could be an important step in the development of microbial strategies to help plants increase their stress tolerance. Such strategies may include the discovery and targeted inoculation of new PGP microbes into agricultural fields [48] or active management of soil communities through agricultural practices that support the presence or high abundance of important root microbiome members that promote plant abiotic stress tolerance [49]. However, determining how root-associated microbial communities respond to abiotic stress conditions is notoriously difficult because a plant's root microbial community will reflect the direct effects of abiotic stress on the bulk soil, as well as the indirect effects of plant responses — e.g. via changes in root structure or exudates. Further complicating the situation, we still do not fully understand the relative contributions and complex interactions between the soil and plant factors that produce the resultant root microbial communities [50].

Here, we consider the impacts of water stress, specifically flooding and drought, and changes in nutrient availability, specifically N and P deficiency stress, on bulk soil microbial communities and plants that shape the root microbiome (Figure 1). We primarily focus our discussion on responses of root bacteria communities, owing to the larger body of research available, but we also briefly mention the effects on fungal communities when possible. For consistency, when discussing bulk soil microbial communities, we include studies examining unplanted soil samples, or those where a concerted effort was made to collect soil outside the influence of plant roots, except when noted. However, the presence of roots may confound some conclusions. When considering effects on root communities, we include studies where at least one of the aforementioned root compartments was sampled and specify the compartment when possible. However, there is considerable ambiguity in defining what constitutes these compartments and variation in the experimental methods used to sample them [5]. This necessitates the use of an all-encompassing term, and thus when discussing these compartments generally, we refer to ‘root-associated’ communities or the ‘root’ microbiome. For both environments, we synthesize the general responses of microbial diversity and community composition to the investigated abiotic stress factors and provide an overview of some of the known mechanisms driving the reported trends. We conclude by identifying areas of future research needed to tease apart the effects of abiotic stress on the soil, plants, and their associated microbial communities.

## Flooding

Water is necessary for life, but most land plants, particularly crops, are negatively impacted by long-term inundation by water [51,52], and the frequency and severity of floods are expected to increase with global climate change. Submergence of non-photosynthetic plant tissues like roots results in a decline in oxygen (O_2_) levels, inhibiting cellular respiration, and is one of the most severe stresses plants face. Additionally, many biochemical and physiological changes occur in plants in response to flooding, most notably a rapid increase in the production of the plant stress signaling hormone ethylene which negatively affects shoot and leaf morphology (Figure 1) [51,53]. A better understanding of how microbial communities respond to flooding is important for the potential discovery of PGP bacteria or fungi that may help confer stress tolerance to plants faced with hypoxic or anoxic growth conditions. As an example, many bacteria have been demonstrated to modulate ethylene levels in plants by producing the enzyme 1-aminocyclopropane-1-carboxylate (ACC) deaminase. ACC is the plant-produced, immediate precursor to ethylene. Bacteria producing ACC-deaminase cleave ACC and reduce the amount of ethylene plants can biosynthesize, thereby reducing plant damage under stress [54,55]. This has been shown in basil (*Ocimum sanctum*) where plants inoculated with ACC-deaminase producing bacteria benefited from increased growth and lower ethylene levels under waterlogged soil conditions compared with uninoculated plants [56]. Similarly, proteomic profiling of cucumber (*Cucumis sativus*) inoculated with ACC-deaminase producing *Pseudomonas putida* UW4 and grown under hypoxic conditions revealed a shift in the protein profile towards proteins involved in nutrient metabolism, defense stress, and antioxidant activity, possibility explaining the growth-promoting mechanisms of the bacteria [57]. Despite this demonstrated potential of plant-beneficial microbes under flooded conditions, it appears only very few studies have characterized root-associated microbial community responses in non-wetland plants species exposed to flooding, with little emphasis placed on crop species; all have focused exclusively on changes in bacterial communities. For example, terminal restriction fragment (T-RF) length polymorphism profiling of root-free bulk soil, rhizosphere, and whole root (rhizoplane and root endosphere) samples from poplar (*Populus* sp.) seedlings subjected to an experimental flood revealed a strong shift in bacterial community composition in the rhizosphere and whole root compartments, compared with bulk soil [58]. This was attributed to a marked increase in the relative abundance of a T-RF likely from the bacterial genus *Aquaspirillum* [58]. Similarly, in a study of wheat (*Triticum aestivum*), a combination of flooding and N limitation decreased the abundance of certain denitrifying bacteria and altered their community structure in the rhizosphere compared with bulk soil samples collected outside the physical influence of roots [59].

The soil in which plants grow undergoes many physicochemical changes in response to oversaturation by flooding. Soil pores, which normally permit gas exchange between the atmosphere, soil, and soil microorganisms, become filled with water and gaseous diffusion is greatly reduced [60]. Soil O_2_ levels can quickly become depleted by aerobic microbes, reaching anoxia even in the uppermost bulk soil layers within hours of a flooding event (Figure 1) [61]. This change in O_2_ availability can then result in a progressive shift in the microbial community from aerobic organisms, to facultative anaerobes, and finally to strict anaerobes [62]. This shift toward anaerobic bacteria was hypothesized to be one possible explanation behind the increase in the relative abundance of *Aquaspirillum* in flooded poplar rhizosphere and root samples, as the genus contains a few known anaerobic species [58,63].

Many plants respond to flooding by forming specialized, gas-filled tissues known as aerenchyma which facilitate the transfer of O_2_ from the still oxic shoots to anoxic roots (Figure 1). Such tissue is well developed in aquatic and wetland plants, but in non-wetland plant species it is typically only formed as a stress response to environmental conditions, like flooding, and has been observed in important crop species like rice (*Oryza sativa*), barley (*Hordeum vulgare*), maize (*Zea mays*), wheat [64,65], soybean (*Glycine max*) [66], and sugarcane (*Saccharum* spp.) [67]. O_2_ diffused to roots via aerenchyma is either consumed by plant cells during respiration or can diffuse from roots to the rhizosphere in a process known as radial oxygen loss (ROL) (Figure 1). ROL can create aerobic conditions in the rhizosphere, theoretically shifting the composition of rhizosphere communities compared with the still anoxic bulk soil by supporting populations of heterotrophic, methane-oxidizing, and nitrifying bacteria, all of which require O_2_ as an electron acceptor for respiration [60,68]. The dimensions of this oxygenated rhizosphere have been shown to be spatially and temporally heterogenous, being dependent on plant species, age and radius of the root, and O_2_ uptake rate of the soil [69], which, in turn, is influenced by soil microbial respiration and oxidation of soil ions like Fe^2+^, Mn^2+^, NH_4_^+^, and S^2−^ [70]. Estimates of this oxygenated zone have primarily been calculated for rice and range from 1 to 3 mm from the root surface [69,71]. However, ROL appears to be context dependent, as Hamonts et al., [59] found a strong decrease in wheat rhizosphere O_2_ concentration upon flooding that persisted throughout the length of the experiment. They hypothesized that shifts in the denitrifying bacterial community were the result of the combined effects of O_2_ and N stress on the plant, which may have reduced root C exudation, one of the most important factors affecting denitrifying activity in soil [59,72]. Although some evidence supports altered exudation of total organic carbon in plants exposed to flooding [73], changes in root exudates from flooded non-wetland species and consequent effects on root microbial communities remain relatively unexplored.

Plants have also been reported to secrete phytotoxic compounds like ethanol, lactic acid, and alanine, which build up in root tissue in response to aerobic respiration under low O_2_ conditions, in an attempt to prevent cell damage caused by flooding [51,74]. This has been demonstrated in tomato (*Lycopersicon esculentum*) [75], pea (*Pisum sativum*) [76], maize [77], and many tree species [78]. However, possible effects of exudation of phytotoxic and other compounds on root-associated bacterial communities also remain unexplored. Graff and Conrad [58] hypothesized that the previously mentioned increase in relative abundance of *Aquaspirillum,* a bacterial genus with known ethanol catabolizing species [63], in flooded poplar rhizosphere and root samples could be due to the exudation of ethanol from roots, a known oxygen stress tolerance mechanism of poplar, as well as to the decrease in O_2_ availability.

Taken together, the above studies demonstrate that root-associated microbial communities can, theoretically, be shaped by changes in O_2_ availability resulting from soil respiration and ROL from plant roots under flooding, as well as changes in exudates, evidence that root-associated microbial communities are shaped indirectly by plant responses to flooding. However, the magnitude of ROL in plant species other than rice is poorly characterized and perhaps context dependent. Thus, increased focus on the response of agricultural plant species to flooding appears warranted, particularly given the threat of future flooding events in agricultural ecosystems. Moreover, continued work is needed to fill the knowledge gaps in how root exudation changes when a variety of crop species are faced with flooded growth conditions, how these exudates shape both bacterial and fungal community diversity and composition in roots, and the consequences for plant growth and functioning.

## Drought

While the precise definition of drought is often debated and depends on historical precipitation data for a defined area and time period [79], here we discuss any circumstances in which a lack of water causes stress to plants. Compared with flooding, considerably more work has focused on the effects of drought as a water stress factor shaping soil and root-associated microbial communities. For a more in-depth discussion of these patterns, we direct the reader to the recent review of Naylor and Coleman-Derr [50].

Although levels of root colonization by AMF have generally been shown to decline under drought [80,81], AMF can promote plant drought fitness either via enhanced nutrition or more direct effects on stomatal conductance and enhanced water use efficiency, but the results are often AMF species specific (extensively reviewed in [81]). Studies characterizing drought-induced changes in non-mycorrhizal, root-associated fungal communities appear to be limited, however, and have concluded that root-associated fungal communities are either unresponsive [82] or exhibit negligible changes [83], possibly because bulk soil fungal communities have also been reported to be generally unresponsive to drought conditions [84–86]. Reported exceptions to this certainly highlight that specific fungal groups may be susceptible to drought [87], and that these communities are shaped by many other factors besides soil moisture content [87–89]. Similarly, Santos-Medellín et al. [90] recently reported substantial root-associated fungal community restructuring in rice as a result of drought exposure, but poor taxonomic resolution meant specific drought-responsive taxa could not be distinguished. As a result, continued research is needed to resolve the responses of specific fungal taxa to drought in both bulk soil and plant roots.

Considerably more work has focused on profiling bacterial community responses to drought, and many recent studies characterizing the root bacteria microbiomes of rice [90], sorghum (*Sorghum bicolor*) [91], and diverse lineages of plant species [92,93] under drought stress have reported enrichment of bacteria from the phylum Actinobacteria. For example, a study of 30 genetically divergent plant species, subjected to drought or watering treatments, revealed the relative abundance of Actinobacteria, particularly the genus *Streptomyces*, in root endosphere communities increased roughly six-fold under drought conditions, although results varied by plant species [93]. Moreover, plant species’ resistance to drought was positively correlated with the increased relative abundance of one sequence variant from the genus *Streptomyces* [93]. Similarly, other *Streptomyces* isolates demonstrated modest PGP activities by increasing root growth in sorghum seedlings [91]. Taken together, these findings suggest a long-conserved, potentially beneficial relationship between a variety of plant species and Actinobacteria [92].

Direct effects of drought on soil bacteria communities could explain the increase in Actinobacteria in the root microbiome of droughted plants, as similar increases in Actinobacteria relative abundance have been reported in bulk soil under drought conditions [50,94,95]. Many members of the Actinobacteria are gram-positive (G^+^), monoderm bacteria generally characterized by having a thick, peptidoglycan cell wall, in contrast with gram-negative (G^−^) bacteria, which are typically diderm taxa with a thinner, more permeable cell wall structure [50,96,97]. As soils become drier, nutrient availability decreases because diffusion pathways moving water-soluble compounds between soil particles and microbes are reduced or disappear entirely, and water potentials decline, placing osmotic stress on microbes [98] (Figure 1). It is thought microbes must accumulate osmolytes inside their cells to lower their internal solute potential and avoid losing water to their environment [98,99]. Gr^+^ bacteria are thought to accumulate osmolytes both on a constitutive and drought-induced basis, increasing their tolerance to osmotic stress under drought [99], and their cell wall layer is thought to improve their drought tolerance by increasing their ability to resist desiccation [97]. Thus, Actinobacteria are physiologically adapted to drought conditions, and this competitive advantage also allows them to thrive in the droughted root microbiome. However, recent evidence highlights the roles plants play in mediating root-associated microbial community responses to drought. For example, Koyama et al. [100] compared microbial communities in bare and vegetated soil samples from plots subjected to drought and different temperature treatments. Interestingly, drought only had a significant effect on bacterial community composition in soils planted with a grassland plant community, and these differences were driven by higher relative abundances of Actinobacteria in droughted soils. Because a similar increase in Actinobacteria was not seen in bare soils, part of the observed increase was likely the result of C rhizodeposition or root metabolites from plants. Fitzpatrick et al., [93] further clarified the relative effects of soil and plants on root-associated community composition under drought by surveying bacterial communities associated with toothpicks placed in the soil as a non-living root substitute. These also exhibited enrichment of Actinobacteria under drought conditions, but these bacteria were not the same as those that were associated with increased drought tolerance in living root samples. This would suggest that the presence and abundance of certain Actinobacteria lineages in the root microbiome under drought results from a combination of direct effects of drought on bulk soil, which favors Actinobacteria generally for their ability to tolerate dry conditions, and indirect effects, via still relatively unexplored metabolic changes in the host plant, which attract specific bacteria that enhance plant drought tolerance.

Plants undergo a variety of morphological and metabolic changes under drought that can then have effects on their root microbial communities, including an increase in the root:shoot ratio, as plants stimulate root growth to access areas of moisture in other parts of the surrounding soil (Figure 1) [50,101]. Plants increase root depth and density and invest in the growth of fine roots, which are the most active root sites in water uptake [102]. Considerable heterogeneity in root-associated microbial community diversity and composition has been demonstrated across root types and lengths within species and genotypes even when plants are cultivated under optimal growth conditions [103–106]. However, experimentally linking drought-induced changes in root morphology to effects on root-associated microbial communities is challenging and has not been attempted.

A commonly reported plant metabolic response to drought is a reduction in photosynthetic activity due to stomatal closure and reductions in enzymes essential for C assimilation and utilization (Figure 1) [107]. This is consistent with recent findings in cottonwood trees (*Populus deltoides*) [108] and a mountain grassland community [101] exposed to drought. Most microbes are C limited, and C provided through rhizodeposition is important in driving microbial processes and shaping root-associated microbial communities [109]. While deposition of newly fixed C from roots might be expected to decrease under drought as consequence of reduced photosynthesis or a shifting of C towards stress-reducing compounds [50], evidence suggests that C exudation from roots may actually increase under drought in a variety of different plant species (Figure 1) [73,110], especially if the drought is mild to moderate [111]. For example, Karlowsky et al. [101] quantified the flow of plant-derived C into the rhizosphere of a mountain grassland community under an experimental drought using atmospheric pulse labeling with highly enriched ^13^CO_2_. While exudation of newly fixed C to the rhizosphere continued under drought, this did not result in increased recovery of labeled C in microbial growth-related lipid biomarkers, suggesting reduced microbial metabolic activity possibly because of restricted resource diffusion throughout the soil [98]. Moreover, consistent with trends commonly observed in bulk soil microbial communities [50,95] total abundance of biomarkers for Gr^+^ bacteria was higher in drought treatments. Interestingly, total abundance of biomarkers for Gr^−^ bacteria was also higher in drought compared with control, perhaps due to the increased C exudation to the rhizosphere during the early periods of drought. This is in accordance with previous findings showing that Gr^−^ bacteria prefer more labile, plant-derived C [112] and provides further evidence that root-associated microbial communities under drought are shaped by plant-mediated effects.

Drought-induced changes in root metabolites may also explain observed changes in the root microbiome (Figure 1). A shift in relative abundance towards monoderm, primarily Actinobacteria, dominated communities in sorghum grown under drought conditions was correlated with changes in the root metabolomic profile [91]. Droughted roots were significantly enriched in many carbohydrates and amino acids, with the strongest enrichment in droughted roots being the compound glycerol-3-phosphate (G3P), an important precursor to peptidoglycan biosynthesis. Meta-transcriptomic sequencing of rhizosphere samples revealed up-regulation of genes associated with G3P transport, primarily in Actinobacteria, suggesting the plant may produce G3P in response to drought, perhaps to reduce oxidative stress in its cells, which is released into the rhizosphere and opportunistically utilized by Actinobacteria for growth [91]. Beyond simple opportunistic growth, the transcriptional activity of root microbiome members, including known PGP bacteria, has been shown to actively respond to plant stress conditions. For example, Sheibani-Terzerji et al. [113] reported up-regulation of genes likely involved in detoxification of reactive oxygen species (ROS) in the root endophytic bacteria *Burkholderia phytofirmans* PsJN colonizing drought-exposed potato (*Solanum tuberosum*) plants. ROS are partially reduced forms of atmospheric O_2_ that exist naturally in plant tissues and are thought to serve as stress and defense signaling molecules [114]. Under drought conditions, however, ROS production can increase due to altered photosynthesis pathways and reach levels that result in oxidative damage and cell death [107,115]. Up-regulation of oxidative stress response genes could be indicative of a response by the bacterium to prevent cell damage in itself; however, *B. phytofirmans* PsJN was also shown to increase drought tolerance in maize by increasing biomass, chlorophyll content, and relative leaf water content [116]. Thus, the intriguing possibility exists that upregulation of these bacterial genes could be a general characteristic in the bacterium aimed at reducing oxidative stress in its plant host [113]. Interestingly, when inoculated alone into gnotobiotic growth systems, isolates from the actinobacterial genus *Streptomyces* also demonstrated modest PGP activities by increasing root growth in droughted sorghum [91]. Together, these examples suggest that drought-induced metabolic changes in the host plant may partly serve to attract specific root-associated bacterial taxa that enhance drought tolerance and that the transcriptional activity of these members responds accordingly to these metabolic signals.

Drought represents a wide-scale disturbance to soil and plants alike. While general trends in root microbiome responses, particularly for bacteria, tend to reflect those reported for soil — namely an increase in the relative abundance of Gr^+^ bacteria and the enrichment of certain monoderm bacteria, notably from the phylum Actinobacteria — an increasing body of evidence suggests that metabolic and morphological changes in plants under drought exert considerable influence on the composition of their root microbiomes. This underlines the importance of including unplanted soil controls and separate analyses of planted and unplanted samples when trying to differentiate the direct effects of drought on soil microbes from indirect plant-mediated effects. In the future, experiments characterizing the root microbiomes of mutant plants engineered for drought tolerance or naturally drought-tolerant accessions may help to shed light on the roles specific members of the root microbiome play in conferring drought tolerance to plants. Such experiments could serve to discover new potential microbial inoculants to alleviate drought stress in crops. Above, we have highlighted a few examples of PGP root microbiome members, namely in sorghum [91], potato [113], and maize [116]. However, numerous other examples exist including enhanced drought tolerance via increased photosynthesis, evapotranspiration, and stomatal conductance in pepper (*Capsicum annuum*) inoculated with various root bacteria isolated from naturally drought-tolerant plants [117] and increased biomass and chlorophyll content in droughted *Arabidopsis thaliana* (hereafter: Arabidopsis) inoculated with the PGP fungus *Piriformospora indica* [118]. This was attributed to active priming of a diverse set of drought-stress related genes in Arabidopsis leaves [118]. Such microbes represent potential inoculants that could prove to be useful in increasing plant tolerance to drought, but their ultimate success in the field depends on their ability to colonize the plant and overcome the dynamic growth conditions and interactions with other soil and root microbes [119]. Thus, continued genomic characterizations of crop species are necessary to uncover the genetic regulators of root microbiome assembly and function under droughted growth conditions and understand the interplay between host and microbe that controls the PGP mechanisms of bacteria and fungi. Advances in this understanding could result in the development of new crop genotypes bred especially for improved responsiveness to the application of specific microbial inoculants [48].

## Nitrogen

N is a vital element for all life forms and one of the limiting nutrients for plant growth in terrestrial ecosystems [120,121]. As most of the N present in the environment is in an unreactive N_2_ form, technological advances, i.e. the Haber–Bosch process, have been instrumental in allowing humans to supplement BNF. The advent of synthetic N fertilizers has dramatically increased grain and biomass yields in agricultural production [45,122], but overuse of N fertilizers and other anthropogenic alterations of the N cycle result in high N deposition into terrestrial ecosystems, with rates projected to increase [32]. Simultaneously, there are soils that suffer from a N deficit, particularly in sub-Saharan Africa where access to N fertilizer for rural farmers is limited and expensive [123], highlighting inequality in the global the distribution of soil N availability [124]. Thus, N stress could be considered a two-sided stress factor, with some environments exhibiting N limitation and others oversaturation [123]. However, here we primarily focus on the effects of N deficiency stress, particularly within the context of agriculture, which is often experimentally investigated by manipulating N availability in soil via the addition of N fertilizers. The N-fixing abilities of microbes like rhizobia and their association with leguminous plants are well established and extensively reviewed elsewhere [125,126]. However, the possibility that other microbes could play a role in the transformation and provision of N to other plant groups means investigating the effects of changing N availability on soil and, in turn, root-associated microbial communities is important for identifying other beneficial microbes that may enhance plant tolerance to N deficiency.

While AMF community responses to nutrient deficiency are typically considered within the context of their responses to soil P levels, more research is emerging to suggest that AMF also respond to changes in N availability and play a positive role in plant N acquisition [41,127,128]; although the rate of N transfer can vary widely [41]. However, little attention has been placed on characterizing the responses of other, non-mycorrhizal root-associated fungal communities to N deficiency. One notable exception is a study of bulk soil and root-associated fungal community responses to low and high N fertilization in sugarcane [129]. Generally, soil and root-associated fungal communities showed compositional differences, indicative of a plant selection for its root fungal community. However, in both bulk soil and roots, low rates of N fertilization supported an increased relative abundance of fungi from the genera *Clonostachys* and *Resinicium*, both of which have species demonstrating biological antagonism against pathogens [129]. Thus, reduced N availability in soil influences communities of root-associated fungi other than AMF— a conclusion also supported by recent findings in root-associated fungal communities of N-starved wheat [130], and the possibility that these fungi may be implicated in providing plant-beneficial services under low N certainly merits further investigation.

While soil N availability gradients have been demonstrated to have strong effects on determining plant community composition [131], this is not always the case with root-associated microbial community composition, consistent with many soil microbes being C limited. For example, genotype explained more variation in rhizosphere bacterial community composition in *Medicago truncatula* than N fertilization rate [132]. Soil type was more determinative of community composition than N level in a comparison of root-associated bacterial communities in sugarcane grown under standard and reduced N fertilization rates in Australia [133]. This was despite differences in biomass suggesting plant growth was N limited and highlights the role of bulk soil edaphic factors in determining root community composition. Nevertheless, reported responses of root-associated bacteria community composition to changes in N availability, at least at higher taxonomic levels, are similar to those reported for bulk soil. Notably, the relative abundance of Acidobacteria was strongly depleted in wheat rhizosphere communities receiving inorganic N additions, a phenomenon widely reported in bulk soil communities [134–139]. The relative abundance of Bacteroidetes in the wheat rhizosphere strongly increased in response to inorganic N additions [140], a result reported previously in N amended bulk soils [134]. Differences in life-history strategies, i.e. copiotrophic versus oligotrophic, are also reflected in the observed shifts in bacterial community composition in response to N fertilization. As higher N availability is linked with increased plant net primary production [141], C availability in soil can increase in the short term as a result of rhizodeposition [142] and in the long term, particularly in agricultural ecosystems, as a result of increased input of higher quality plant residues into the soil after harvesting [143]. These inputs of labile, organic C combined with increased N availability provide a nutrient-rich environment for microbial growth and may explain the higher relative abundances of phyla, like Bacteroidetes, generally regarded as copiotrophic [144]. Conversely, decreased relative abundances of bacteria from the Acidobacteria under high N is in accordance with the commonly accepted idea that this phylum contains oligotrophic members that have a competitive advantage in soil environments with limited nutrient availability and more recalcitrant C sources [144,145].

Because many of the abovementioned findings are hypothesized to be at least partially explained by changes in the quality or quantity of C inputs resulting from N addition, it is highly likely that the results of many of the studies on soil community responses to changes in N availability have been influenced by plant rhizodeposition. The few studies that have explicitly included unplanted soil controls in their experimental design have indicated that addition of N to bare soil has no effect on bacterial abundance [146], or bacterial community composition [132] compared with unfertilized soils. Although limited in scope, these findings suggest that many of the hypothesized reasons for changes in microbial abundance, diversity, and community composition in the soil are more influenced by indirect plant effects rather than a direct effect of N [132,146,147].

N-deficient plants generally respond by decreasing root growth, reducing photosynthesis, and inducing early leaf senescence (Figure 1) [148]. Other plant responses to low N alter rhizosphere chemistry, including composition and quantity of root exudates, and have concomitant effects on root-associated microbial communities (Figure 1) [73,147,149,150]. For example, axenically grown maize plants exposed to N deficient growth conditions exhibited a lower concentration of many amino acids in their root exudates compared with control plants [149]. Similar findings have been reported for common bean (*Phaseolus vulgaris*) [151] and are consistent with the idea that amino acid stocks are an important indicator of the N status of plants and may trigger plant responses to changes in N availability in soil [152]. Shifts in root exudation profiles have also been hypothesized to drive observed changes in root microbial communities. An increase in bacterial abundance in the barley rhizosphere under high N growth conditions [153], shifts in root endosphere bacteria community structure in sorghum grown in +N or −N conditions [154], and enrichment of certain bacterial families in the root microbiome of N-starved wheat [130] were hypothesized to be at least partly the result of changes in quality and/or quantity of root exudates under different levels of N availability. However, exudates were not specifically characterized in any of these cases.

Other studies have explored the link between altered root exudation and root-associated microbial community functioning; for example Kavamura et al. [140] reported that rhizosphere bacteria communities of wheat plants receiving no inorganic N fertilizer were enriched for predicted functional pathways related to terpenoid metabolism and depleted in genes related to metabolism of amino acids and carbohydrates. Terpenoids are plant root exudates and known nitrification inhibitors [155,156], which could potentially mitigate N loss by nitrification under low N conditions. However, it is still unclear if terpenoid release is an active plant adaptation strategy to nitrifying growth conditions, or if they serve another, still unknown, purpose with nitrification inhibition as a side effect [155].

Characterizing root exudates from maize grown under progressively increasing N levels, Zhu et al., [150] reported that total root exudation of sugars, sugar alcohols, and phenolic compounds was positively and significantly correlated with increasing N levels. This drove differences in bacterial community composition in addition to differences in 16S rRNA gene copy number per gram of rhizosphere soil, suggesting changes in root exudation under high N increase bacterial abundance in the rhizosphere. Additionally, the relative abundance of certain nitrifying and denitrifying genes, predicted by 16S rRNA gene sequences, and total abundance of N cycling genes, significantly increased with higher N application. This finding was coupled with reduced N recovery and higher rates of fertilizer N loss in the high N treatment, suggesting changes in root exudation patterns shifted rhizosphere community activity in a way that contributed to reduced N use efficiency [150]. Finally, transcriptomic sequencing methods have permitted higher resolution characterization of changes in active metabolic processes of root microbiome members in response to N availability induced changes in root exudation. Carvalhais et al. [157] incubated pure cultures of the known PGP bacteria *Bacillus amyloliquefaciens* FZB42 with root exudates from maize plants grown under N-deficient conditions. Transcriptomic sequencing revealed down-regulation of bacterial genes thought to be involved in protein synthesis, a general indication of a stress response possibly caused by reduced amino acid availability, the presence of antibiotics, or other growth inhibitors in the root exudates. Moreover, since the demonstrated PGP abilities of *B. amyloliquefaciens* FZB42 are related to enhanced P, but not N, acquisition, significant down-regulation of bacterial genes involved in chemotaxis and cell motility suggested that maize exudates under low N conditions may be involved in inhibiting root or rhizosphere colonization of microbes that may compete with the plant for scarce N resources [157].

The findings of the work discussed above highlight the strong indirect effects of plant responses to N availability, primarily through changes in root exudate quantity and composition, have on shaping the abundance, composition, and metabolic functioning of the root microbiome. Moreover, plants may not only recruit beneficial microbes to their roots that improve N acquisition, but also actively discourage colonization by microbes that provide them no benefit or represent a competitive threat in their current growth environment. In the future, experimental approaches that aim to decouple the direct effect of N availability on microbial communities from effects of altered root exudation will be key to further unraveling the complex effects of N stress on root microbiome assembly, composition, and functioning. Such future work should include continued characterization of root exudation profiles of a variety of plant species grown in N limited conditions coupled with transcriptomic profiling to identify possible candidate genes involved in regulating plant responses to N limitation.

## Phosphorus

The availability of P is another factor limiting primary plant productivity and cellular biological processes [158,159]. P does not exist in any significant quantity in a gaseous form [47], and the only source of atmospheric P into soil comes from relatively minor inputs of mineral aerosols [160]. Although P is naturally abundant in the earth's crust, plants can only uptake phosphate, in which most soils are deficient [161]. Moreover, much of this phosphate is present in the form of highly concentrated phosphate rock [47], which weathers slowly and is not generally considered a viable input source to replace biologically depleted P [158]. Additionally, most of the inorganic and organic P present in the soil is adsorbed to soil minerals or immobilized in biomass or other organic matter [162]. As such, particularly in agricultural ecosystems, P availability in soil is increased through the addition of fertilizers produced from the acid extraction of mined P rock. As global agricultural demand increases, the quality and availability of easily mineable rock for fertilizer production continue to decline [46,47], heightening interest in strategies to make crops more tolerant of P deficiency. While microbes can increase the ability of plants to uptake P from the soil, either by root extension and growth stimulation or by solubilizing P into plant-available forms, they are also P limited and must effectively compete with plants for scarce P [162]. Thus, understanding how soil and root-associated microbial communities respond to P replete or deficient conditions is an important step in identifying which microbes are P stress tolerant and may be able to provide P acquisition services to a host plant.

Root bacterial microbiome responses to changes in P availability vary from modest shifts in community structure in Arabidopsis [163] to no effects in switchgrass (*Panicum virgatum*) [164] and ryegrass (*Lolium perenne*) [165]. In maize, P fertilization increased rhizosphere bacterial diversity and shifted bacterial community composition [166], and changes in P availability, as a result of P fertilization, were a major driver of bacterial community structure in rhizosphere and root endosphere samples, explaining more variation than plant genotype or differences between bulk soil and root samples [167]. Mander et al., [168] reported a higher frequency of P solubilizing bacteria isolated from the rhizospheres of ryegrass and clover (*Trifolium repens*) growing under low P conditions suggesting a selective pressure for P solubilization based on soil P availability. Similarly, P-limited growth conditions increased the diversity of bacteria with alkaline phosphomonoesterase — a bacterial enzyme involved in the mineralization of insoluble organic P to soluble P [169] — producing genes and shifted bacterial community composition in the barley rhizosphere, whereas there was no similar response in wheat [170]. Together these results highlight the differential microbial responses to P stress among plant species [171], but it is worth noting that these experiments are performed under a variety of conditions that likely vary in baseline P availability.

The low availability of P in most soils limits plant productivity in many ecosystems [172]. To cope, plants have developed many morphological and physiological mechanisms for increasing access to P, termed phosphate starvation responses (PSRs). These PSRs include changes in root morphology, like branching and root hair formation, to better exploit phosphate resources in upper soil layers [173,174], enhanced expression of P transporters [175], and the of release of phosphatases to access organic P and organic acids to mobilize P that is bound in soil (Figure 1) [176,177]. For example, exudation of amino acids has been shown to increase under P-deficient conditions in pine (*Pinus radiata*) [178], common bean [179] and in rice [180], and higher root exudation under low P conditions has also been linked to enhanced P uptake in maize and mung bean (*Vigna radiata*) [181]. Such changes in root morphology and root exudation under P stress could also influence diversity and composition of root microbial communities, supporting a conclusion that responses of the root microbiome to P stress are plant mediated.

Recent studies using Arabidopsis have employed targeted genomic approaches to establish more causal links between genetic regulators of PSRs and root microbiome assembly and made considerable progress in demonstrating that root microbiome composition under P stress is plant regulated [182–184]. For example, Castrillo et al., [182] used PSR-mutant Arabidopsis plants, either lacking genes involved in specific phosphate transporters or full transcriptional regulators of PSR, and non-mutant wild-type (wt) plants to show the assembly of the root microbiome is mediated by the plant PSR genotype. PSR mutants developed unique root bacterial microbiomes compared with wt plants even when grown under P-replete conditions. This finding was consistent across plants growing in soil and on agar plates inoculated with a diverse bacterial synthetic community, confirming the importance of PSR regulating genes in determining root bacterial microbiome composition. Moreover, the master transcriptional regulator of Arabidopsis PSRs, *PHR1*, was also demonstrated to be involved in suppressing plant defense responses during PSR. *PHR1* mutants, with reduced PSR expression, showed enhanced tolerance to pathogen infection, suggesting that PSRs and plant defenses are closely linked, and plants with intact PSR regulators prioritize phosphate acquisition over defense in low phosphate growth conditions [182]. Similar results have been reported for root fungal communities, generalizing the findings across both microbial kingdoms. For example, Fabiańska et al., [183] profiled rhizosphere and root endosphere (including the rhizoplane) fungal communities also from wt and PSR mutant Arabidopsis individuals and reported PSR mutants assembled unique fungal root communities compared with wt plants, further confirming that genetic regulators of PSRs play an important role, even under P-replete conditions, in mediating plant–microbe interactions in roots. Most recently, Finkel et al., [184] grew wt and two PSR mutant Arabidopsis genotypes, either lacking a single gene encoding a protein involved in trafficking of high-affinity phosphate transporters (*phf1* mutant) or two genes encoding partially redundant PSR transcription factors (*phr1 phl1* double mutant), in soil collected from fields under different levels of long term P fertilization. Regardless of P availability, the composition of the bulk soil and root bacterial communities was strongly correlated, whereas this was not the case for the soil and root fungal communities, indicative of greater plant selectivity for fungi [184]. Moreover, the effect of the PSR mutant genotype was greater than P availability, suggesting that plant PSRs mediate composition of the bacterial and fungal communities in the root microbiome more than P status [184]. It was hypothesized that PSR regulation in the plant host influences the root microbiome in a combination of two ways. First, PSRs might serve to attract beneficial microbes to the roots that aid the plant in tolerating P stress. Second, the link between genetic PSR regulators and plant immunity demonstrated in Castrillo et al. [182] raises the possibility that the composition of the root microbiome also reflects opportunistic microbes that exploit the plant's compromised immunity under active expression of PSRs [184].

Plant PSRs have also been linked to changes in plant-AMF symbioses, and considerably more work has focused on investigating changes in AMF communities in response to changes in P availability compared with the previously discussed stress conditions. A wide body of experimental evidence has shown that AMF relative abundance in roots based on DNA sequencing and levels of root colonization quantified by microscopy depends on soil P availability, but the results can be species dependent. For example, AMF colonization or relative abundance in roots declined along an increasing soil P availability gradient in petunia (*Petunia* × *hybrida*) [171], pea [185], maize [167,186,187], wheat [188], and across a wide variety of other AMF-associating crop species [189] but not in soybean [186]. Thus, the general pattern that AMF root colonization levels are lower when P availability is sufficient, and the fungal symbiosis is likely not beneficial for the plant suggests active plant mediation of this symbiosis [39]. The factors regulating host-AMF symbiosis under changes in soil P availability are highly complex, and while the specific mechanisms of host-AMF signaling of are beyond the scope of this review and extensively covered elsewhere (see [190,191]), PSR-induced changes in root exudation are thought to play a major role. For example, it was hypothesized that an increase in root exudation of carbohydrates in maize plants grown under P-deficient conditions may be a plant strategy to stimulate growth and activity of AMF [149], but other root metabolites and compounds hypothesized to play a role in regulating the plant-AMF symbiosis have been demonstrated to change depending on soil P availability. In a comparison of root metabolite profiles from pea grown in high or low P conditions, Laparre et al. [192] used liquid chromatography — mass spectrometry to identify 28 ions representing unknown root metabolites that were more abundantly produced under low P growth conditions and hypothesized that these metabolites may play a role in regulation of the AMF symbiosis. Root metabolites, particularly a type of plant hormone called strigolactones, are important in initiating hyphal branching and subsequent root colonization by AMF under normal growth conditions [193–195]. However, an experiment by Balzergue et al. [185] demonstrated that plant strigolactone production is negatively affected by increasing P supply. Growing pea under low or high P availability, the authors reported significantly lower AMF root colonization in the high P treatment as well as a lack of strigolactones in root exudates. Interestingly, when exogenous strigolactones were added to the high P treatment, this failed to stimulate AMF colonization of the plants, suggesting that other signaling mechanisms must also play a role [185]. These findings highlight avenues for future research, namely, to elucidate the genetic and chemical drivers of AMF colonization under differences in P availability and continued exploration of the other roles strigolactones may play in plant PSRs.

Plant PSRs have also been demonstrated to regulate the activities of non-mycorrhizal fungi. For example, Hiruma et al. [196] demonstrated the PGP properties of the non-mycorrhizal root endophytic fungus *Colletotrichum tofieldia* in Arabidopsis were the result of increased phosphate translocation into colonized plant roots. Interestingly, this growth promotion effect was only observed under P-deficient growth conditions and disappeared entirely in *phf1* mutant and *phr1 phl1* double mutant plants. Moreover, fungal biomass was higher in *phf1* and *phr1 phl1* mutants compared with wt individuals, suggesting that genetic regulators of Arabidopsis PSRs are involved in limiting the proliferation of the fungus in the roots. PGP activities of *C. tofieldia* were also linked to intact pathways of the Arabidopsis immune system, namely the biosynthesis of indole glucosinolate, with the fungus exhibiting pathogenic characteristics in mutant plants lacking genes involved in this pathway. Together, this highlights an active plant role, through coordination between PSRs and the plant immune system, in regulating the presence and function of certain root microbiome members under P stress conditions [182,196].

The considerable amount of work undertaken characterizing root microbial communities in response to P stress highlights the role of genetic regulators of plant PSRs in shaping the root microbiome. Many of the abovementioned studies have utilized Arabidopsis because of its short generation time, sequenced genome [197], and characterized root microbiome [13]. These advances have permitted identification and manipulation of specific genes that can be used to test hypotheses about plant metabolic processes and changes in root microbiome assembly. While such studies certainly increase our understanding of the genetic and molecular cues dictating microbiome assembly under low P availability, future work should focus on extrapolating these results to agriculturally relevant plant species. Main foci should include identifying the genes and complex metabolic pathways involved in P responses in agricultural crops, development of mutant lines for experiments that investigate the interplay between altered PSRs and root microbiome development, and the breeding of cultivars with enhanced tolerance to low P conditions.

## Concluding remarks

Here, we have synthesized the major effects of water and nutrient stress on bulk soil microbial communities and plants and how these ultimately shape the composition of the root microbiome (Figure 1). Given the close association of plants and their microbial symbionts, it is unsurprising that much of the work discussed here supports a conclusion that plant responses to abiotic stress conditions strongly shape root microbial communities. Perhaps the clearest evidence for this comes from the relatively few studies that have included unplanted soil samples in their microbial surveys and reported no or relatively minor changes in soil microbiome composition compared with root communities under abiotic stress conditions [132,146,163,198]. Thus, the inclusion of unplanted soil samples subjected to the same experimental conditions as those containing plants is a simple, but straightforward technique for separating direct effects of abiotic stress on microbial communities from plant-mediated effects. However, it is important to note that water and nutrient dynamics will differ inherently between planted and unplanted soils, particularly when confined to pots. Therefore, careful experimental planning and execution is necessary to maintain the same conditions in unplanted treatments compared with those experienced by plants

Despite the recognized importance of root exudates in shaping the root microbiome [74,199,200], the results of many of the studies presented here highlight the lack of research focused on characterizing root exudates from a variety of crop species under abiotic stress conditions. This is certainly due to the enormous challenge of collecting root exudates from a native soil environment [201]. These difficulties are further exacerbated by measurement challenges in that there are hundreds of chemically complex primary and secondary metabolites exuded from roots, plants themselves can reabsorb a variety of compounds from the soil, and microbes also release compounds into the soil to stimulate plant root exudation [152,201]. Thus, this close, two-way relationship between plants and their root microbiome makes pinpointing the source and mechanisms of exudate release difficult. In light of these challenges, continued work is certainly warranted to develop new techniques for characterizing and quantifying the root exudates of agriculturally relevant plant species under both optimal and stressed growth conditions over time, and presents unique opportunities to develop advances in experimental tools like exudate sampling systems, isotopic and fluorescent labeling, imaging techniques, and improved mathematical modeling of exudate processes [201].

Many of the abovementioned studies have primarily characterized responses of microbial composition and diversity to abiotic stress conditions using older molecular tools or taxonomic surveys using sequencing of bacterial and fungal marker genes. Although these approaches have been key in revealing the vast diversity of microbial communities, developing a deeper understanding of how abiotic stress influences the interactions among soil, plants, and their microbiomes requires advances in experimental approaches and new tools that move beyond descriptions of taxonomic diversity, particularly when trying to identify and capitalize on the plant-beneficial aspects of the root microbiome for the benefit of agriculture [202]. Here, we have included some examples of and advocated for continued utilization of metagenomic and transcriptomic sequencing techniques to reveal changes in the functional potential as well as gene transcription profiles in both microbes and plants under abiotic stress. Together, these ‘-omics’ techniques are valuable tools for future experiments that aim to manipulate multiple components to uncover the genetic, molecular, and biochemical mechanisms that mediate plant–soil interactions [203].

To achieve such a goal, hybrid experimental approaches that combine existing and developing genomic technologies and classical microbiology techniques will be required [204]. For example, genome editing tools like the CRISPR-Cas9 system now permit the development of mutant lines of important crop species like rice and wheat [205] so that hypotheses about the roles certain genes play in regulating plant responses to abiotic stress can be experimentally tested. Recent advances in single-cell transcriptomic sequencing methods, like Drop-seq, [206] allow for the identification of differences in gene expression between individual root cell types in response to changes in growth conditions [207]. Additionally, there has been a resurgence in interest in microbe collections isolated from plant roots to design synthetic microbial communities that can be inoculated under controlled experimental conditions in microcosms to test hypotheses about plant–microbe interactions [48,119,208]. This approach has been used in investigating the link between plant immunity responses, root exudates, and root microbiome assembly [209] and bacteria-mediated plant phenotypic responses under abiotic stress [210] in Arabidopsis*.* Future experiments with mutant, agriculturally relevant species and microbe culture collections would shed light on the roles specific plant or microbial genes play in assembling or stabilizing the root microbiome and its reproducibility under abiotic stress conditions or reveal root cell-specific transcriptional responses to the abiotic stress conditions discussed here. Moreover, other experimental techniques like split-root pot experiments could prove useful in uncoupling interplant variation in plant stress responses or exploring temporal variability in root microbiome assembly and composition in plants simultaneously subjected to combinations of abiotic stressors or varying levels of stress severity.

Taken together, continued work employing systematic combinations of plant and microbial genomic and metabolomic profiling methods in concert with hands-on manipulation experiments will be instrumental to untangling the complex molecular and biochemical interactions between plants and soil under abiotic stress in controlled conditions and, in time, dynamic conditions in the field.

## Abbreviations

ACC: 1-aminocyclopropane-1-carboxylate
AMF: arbuscular mycorrhizal fungi
BNF: biological nitrogen fixation
C: carbon
G^−^: gram-negative
G^+^: gram-positive
G3P: glycerol-3-phosphate
N: nitrogen
O_2_: oxygen
P: phosphorus
PGP: plant growth-promoting
PSR: phosphate starvation response
ROL: radial oxygen loss
ROS: reactive oxygen species
T-RF: terminal restriction fragment
wt: wild-type
